# Genome architecture plasticity underlies DNA replication timing dynamics in cell differentiation

**DOI:** 10.3389/fgene.2022.961612

**Published:** 2022-09-02

**Authors:** Wenjun Yu, Quan Zhong, Zi Wen, Weihan Zhang, Yanrong Huang

**Affiliations:** ^1^ Center for Genetics and Developmental Systems Biology, Department of Obstetrics and Gynecology, Nanfang Hospital, Southern Medical University, Guangzhou, China; ^2^ Hubei Key Laboratory of Agricultural Bioinformatics, College of Informatics, Huazhong Agricultural University, Wuhan, China

**Keywords:** replication timing, 3D genomics, Hi-C, embryonic stem cell, compartment, TADs

## Abstract

During the S-phase of eukaryotic cell cycle, DNA is replicated in a dedicatedly regulated temporal order, with regions containing active and inactive genes replicated early and late, respectively. Recent advances in sequencing technology allow us to explore the connection between replication timing (RT), histone modifications, and three-dimensional (3D) chromatin structure in diverse cell types. To characterize the dynamics during cell differentiation, corresponding sequencing data for human embryonic stem cells and four differentiated cell types were collected. By comparing RT and its extent of conservation before and after germ layer specification, the human genome was partitioned into distinct categories. Each category is then subject to comparisons on genomic, epigenetic, and chromatin 3D structural features. As expected, while constitutive early and late replication regions showed active and inactive features, respectively, dynamic regions with switched RT showed intermediate features. Surprisingly, although early-to-late replication and late-to-early replication regions showed similar histone modification patterns in hESCs, their structural preferences were opposite. Specifically, in hESCs, early-to-late replication regions tended to appear in the B compartment and large topologically associated domains, while late-to-early replication regions showed the opposite. Our results uncover the coordinated regulation of RT and 3D genome structure that underlies the loss of pluripotency and lineage commitment and indicate the importance and potential roles of genome architecture in biological processes.

## Introduction

One of the most fundamental and interesting questions in biology is how the same genome in embryonic stem cells (ESCs) gives rise to functionally highly diversified cell types through differentiation and lineage commitment. Cell differentiation is a complex program regulated at multiple cellular and molecular levels. Despite significant progress in characterizing changes in the epigenome during cell differentiation (Moore et al., 2020), the interplay of regulation among the levels of genomic sequences, histone modifications, and three-dimensional (3D) chromatin structure remains poorly understood (Soshnev et al., 2016).

The past decade has witnessed remarkable progress in characterizing 3D chromatin architecture with the introduction of novel technologies based on genome-wide chromatin conformation capture (Fullwood et al., 2009; Lieberman-Aiden et al., 2009). A hierarchy of chromatin structure organization across multiple orders of magnitude in size, from A/B compartments (Lieberman-Aiden et al., 2009) to topologically associated domains (TADs) (Dixon et al., 2012; Hou et al., 2012) and loops (Fullwood et al., 2009; Rao et al., 2014; Zheng et al., 2019) have been revealed. Specifically, compartments are represented as a plaid pattern in the heatmap of the interaction matrix, indicating two structurally globally separated components (A and B). The definition of the active (A) and inactive (B) compartments is based on principal component analysis and were first established by Lieberman-Aiden (Lieberman-Aiden et al., 2009). They calculated the correlation of the observed vs. expected Hi-C matrix and used the sign of the first eigenvector to denote the positive and negative parts as A and B compartments, respectively. These compartments are correlated with chromatin states such as gene density, DNA accessibility, GC content, replication timing, and histone marks (Lieberman-Aiden et al., 2009; Pal et al., 2019). The A compartments are more specifically defined to represent the gene-dense regions of euchromatin, while the B compartments represent heterochromatic regions with fewer gene activities. TADs appear as squares along the diagonal of the heatmap, within which chromatin interactions occur more frequently than outside. The promising findings recently motivated the 4D Nucleome project (Dekker et al., 2017) toward deeper mechanismic understandings of nucleus architecture and how its changes affect various diseases (Dudchenko et al., 2017).

Eukaryotic cells duplicate their genomes in a defined temporal order known as the replication timing (RT) program (Marchal et al., 2019). During stem cell differentiation, the order changes in a highly cell type–specific fashion, often correlated with changes in gene and epigenetic activity and subnuclear position (Hiratani et al., 2010; Dileep et al., 2015; Rivera-Mulia et al., 2015). The changes happen primarily in 400–800-kilobase (kb) units designated replication domains (RDs) (Ryba et al., 2010). In the last decade, RT has been extensively measured genome-wide and found to correlate with epigenetic marks (Hiratani et al., 2010) and 3D genome organization including A/B compartments (Ryba et al., 2010) and TADs (Pope et al., 2014). Specifically, both A compartments and early-replicating regions correspond to actively transcribed open chromatin, while B compartments and late-replicating regions correspond to silent compact chromatin. Moreover, A and B compartments are highly correlated with early- and late-replicating DNA, respectively (Ryba et al., 2010; Dileep et al., 2019; Miura et al., 2019). Collectively, RD boundaries align well with TAD boundaries, indicating the consistency of these two types of modular organization in RT and chromatin architecture (Pope et al., 2014).

Despite the intertwined correlations between RT, genome structure, and chromatin marks, recent discoveries on the 3D chromatin architecture hierarchy layer adds another level to the complexities (Gorkin et al., 2014). Here, we collected available relevant data for five human cell types—hESCs and the other four differentiated cell types—and characterized the genomic features, histone modifications, and 3D genome structure underlying the RT program in differentiation. Focusing on the RT-constitutive and RT-switching regions, we identified chromatin features unique to the RT-switching region, which may facilitate lineage specification.

## Materials and methods

### Cell types and datasets

The five ENCODE cell lines used in this study are hESC (cell line BG01) and four lineage-committed cell lines (NHEK, IMR90, K562, and HUVEC) as representatives of three germ layers. The choices of cell type are based on the availability of all the data types required for this study. The GSM accession IDs are listed in Supplementary Table S1.

### Repli-seq data analysis

Repli-seq data of the five cell types were downloaded from ENCODE (ENCODE-Project-Consortium, 2012). The replication time scores were calculated as described previously (Marchal et al., 2018), with slight modification. Based on the clustering result of the four phases (S1–S4) (Supplementary Figure S2), the sequencing data for S1 and S2 were combined. Similarly, S3 and S4 were also combined based on their high levels of consistency. We then calculated the signal score of the sample in100-kb windows after removing chromosomes Y and M as follows.

$$RT=log_{2}\left(\frac{S1+S2}{S3+S4}\right)$$

Positive RT scores represented early replication timing, while negative RT scores corresponded to late replication. We used the limma package in R to normalize and smooth the data and then used the DNAcopy package to obtain the replication domain position at 50 and 100 kb resolution (Ryba et al., 2011). Supplementary Figure S3 summarizes the RT samples used in this study and provides an example. We divided the RT regions into four types (CE, CL, EtoL, and LtoE). The RT value of the CE region was positive in hESC and the four differentiated cell types. The CL region was negative in hESC and the four differentiated cell types. The RT scores of the dynamic EtoL region were positive in hESC and negative in the four differentiated cell types. The RT scores of the LtoE region were negative in hesc and positive in the four differentiated cell types.

### ChIP-seq data analysis

Before mapping, adapter-sequence trimming and removal of low-quality reads were performed using Trimmomatic 0.36 (Bolger et al., 2014). The fastq files were aligned to the human genome (UCSC hg19) using the default settings in Bowtie2 (Langmead and Salzberg, 2012) and retaining only unique alignments. Peaks were called using MACS2 (V2.1.1) (Zhang et al., 2008) with default settings. Due to the different sizes between the ChIP-seq peaks and replication domains, the ChIP-seq signals were processed for downstream analysis as follows. From the histone modification signals in the gene bodies, enhancers, promoters, and open chromatin regions, wecalculated the sums of the signals for each sample in100-kb bins. The input data were subtracted from the normalized ChIP data to obtain an accurate histone modification signal. To define the open chromatin regions, we aligned DNase-seq data to the reference genome, retained the uniquely aligned reads, and called peaks under the MACS2 default parameters for the whole genome. Based on comparisons of Spearman’s correlation coefficients between different types of histone modification signals and replication timing, we used the ChIP-seq signals in the open chromatin regions in the downstream analysis. We used Deeptools to convert the Bam files into Bigwig files to visualize the histone modification signals in Integrative Genomics Viewer.

### Hi-C data analysis

Read pairs were mapped using the juicer pipeline (Durand et al., 2016). To obtain the A/B compartment profiles, we first used the distance-normalized Pearson correlation matrix (bin Size = 500 kb) to calculate the eigenvector values. We then identified the A/B compartments according to corresponding gene expression levels and gene densities. If these values were negatively correlated with the eigenvector values, the signs of the eigenvector values were reversed. We then repeated the calculation at 100 kb resolution to obtain higher-resolution compartment partitions to improve the accuracy of the results.

To determine the genome-wide degree of compartmentalization (Dileep et al., 2015), we used 100-kb binned Hi-C matrices. For each bin, the degree of compartmentalization was calculated as the log ratio of the sum of contacts to the same compartment over the sum of the contacts to the opposite compartment. Thus, a value of 0 indicated that a particular bin interacted equally with early and late compartments.

HiTAD (Wang et al., 2017) is used to identify layered TADs, in which 0 represented TADs of the lowest level in the hierarchy. The larger the number, the higher the TAD level.

### Plotting, clustering, and statistics

The figures in this study were generated using the R package ggplot2. Replication timing was clustered using the R clustering function kmeans. For statistical analysis, we used Wilcoxon rank-sum tests to calculate *p* values by the compare_means function in the ggpubr R package.

## Results

### Genomic characteristics of constitutive and differentiation dynamic replication domains

The human genome was first partitioned into 100-kb bins. According to the RT values in hESC and the other four cell types (HUVEC, IMR90, K562, and NHEK) as representatives of the three germ layers, each bin was categorized as RT-constitutive or RT-switching type. The RT-constitutive regions were further divided into constitutive early (CE) and constitutive late (CL) regions. Among the regions showing RT switching between early and late states, we selected those with early RT in hESC and late RT in all four differentiated cell types, denoted as EtoLs, and late RT in hESC and early RT in all four differentiated cell types, denoted as LtoEs. By requiring consistent RT switching between ES and lineage-committed cell types in all three germ layers, we aimed to identify patterns related to cell pluripotency independent of lineage specificity. Our analysis focused on the four categories of the genomic region; namely, CE, CL, EtoL,and LtoE(Supplementary Figure S1).

**FIGURE 1:**
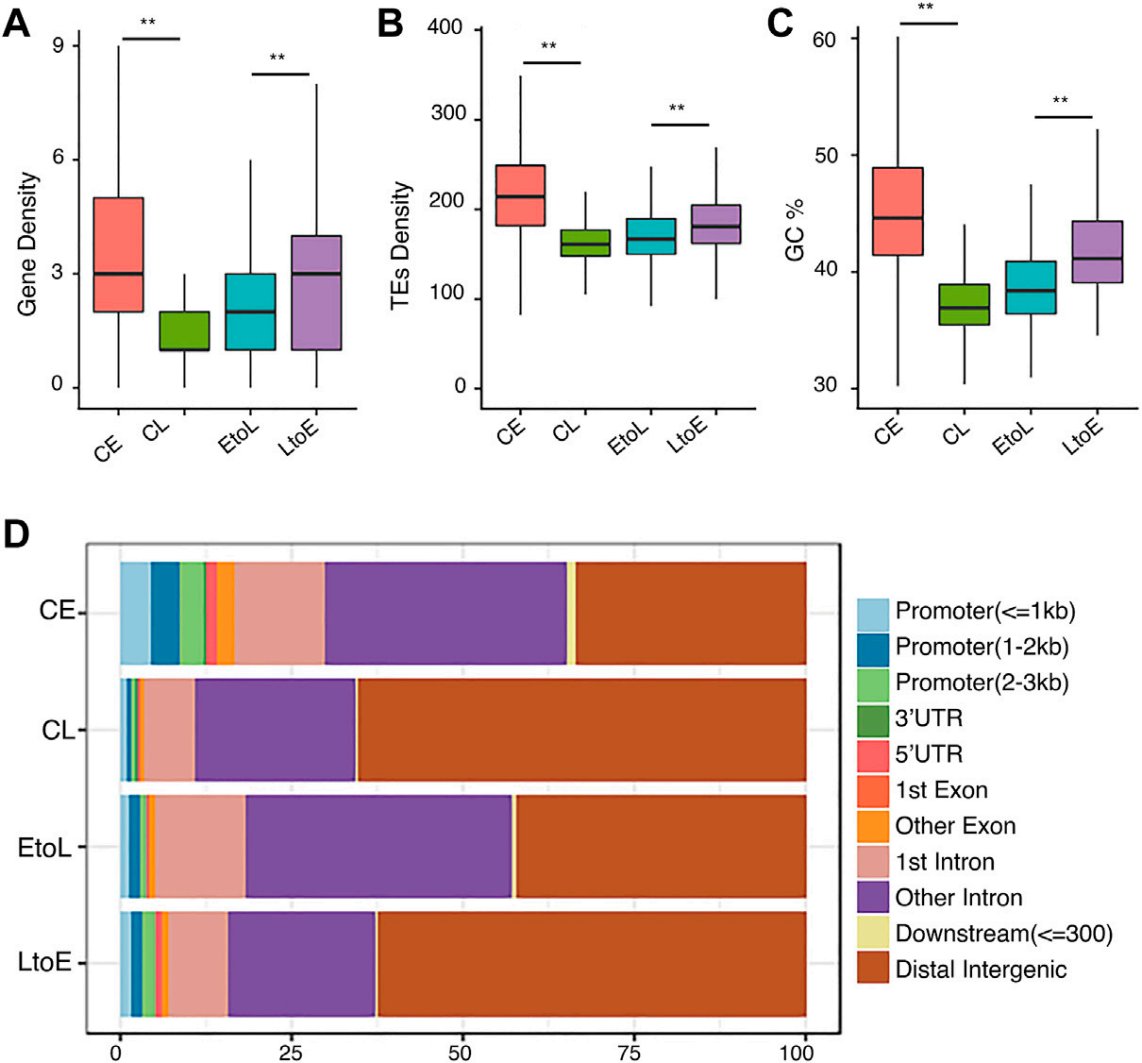
Genomic features of the four replication timing categories.**(A)** Gene density distributions of the four replication domains: constitutive early (CE), constitutive late (CL), early-to-late (EtoL), and late-to-early (EtoL). The unit is the number of genes per100 kb bin.**(B)** Density distributions of transposable elements (TEs).**(C)** GC content distributions.**(D)** Region constitutions with respect to gene structure annotation. *p* values are calculated using Wilcoxon rank-sum tests. **p< 1e-5, ***p< 1e-10.

We analyzed their gene densities, transposable element (TE) densities, GC contents, and genomic annotation-related distributions of the four types of RT regions (Figure 1). Consistent with previous studies (Rivera-Mulia et al., 2015), CE regions showed high gene density, TE density, and GC content, while CL regions showed the opposite. The EtoL and LtoE regions showed intermediate genomic features. These patterns indicated that RT-switching tended to happen in regions with intermediate levels of gene density, TE density, and GC content. Among the two dynamic RT types, LtoE regions showed significantly higher gene density and GC content than EtoL regions. In terms of gene-centric categorization, LtoEs were in more gene-rich regions than EtoLs (Figure 1D). Since different cell lines share the same genome, genomic differences cannot be attributed to RT switching during differentiation. This raises questions regarding what distinguishes the opposite direction of RT switching in dynamic regions.

### Epigenetic features of constitutive and differentiation dynamic replication domains

**FIGURE 2:**
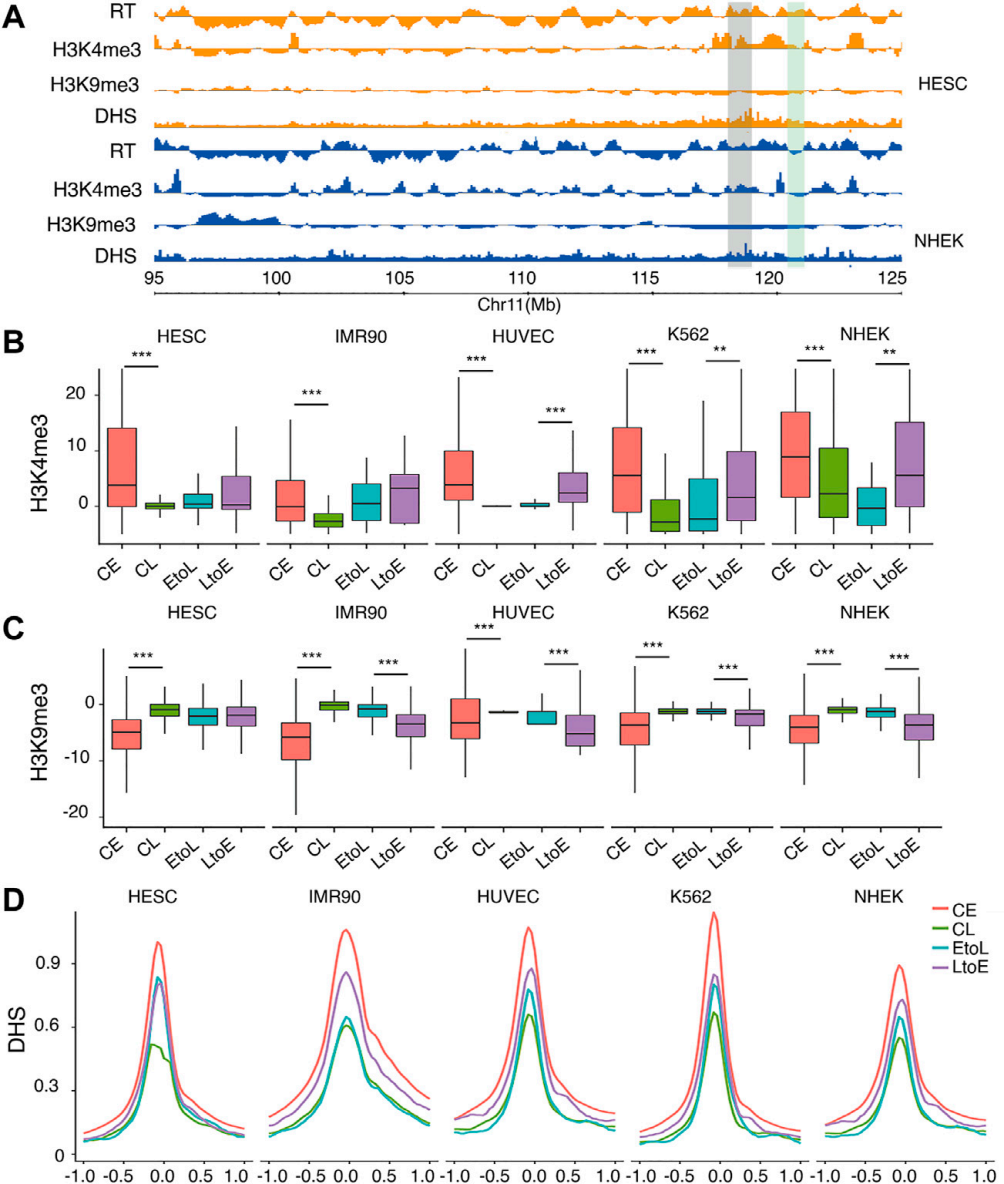
Epigenetic features of the four RT classes.**(A)** Typical region on chromosome 11 showing replicating time, H3K4me3, H3k9me3, and DNase I hypersensitivity sites (DHSs) for hESC and NHEK cells. The region highlighted in gray denotes an area with constitutive early RT. The region highlighted in green denotes an area with RT switching from early to late.**(B)** H3K4me3 signal distribution of the four replication domains: constitutive early (CE), constitutive late (CL), early-to-late (EtoL), and late-to-early (EtoL). The CE and CL regions differ significantly in all cell types. The EtoL and LtoE regions show significantly different signals in differentiated cells.**(C)** H3K9me3 signal distributions. The CE and CL regions differ significantly in all cell types studied. The EtoL and LtoE regions show significantly different signals in differentiated cells **(D)** DHS profile anchored at TSS within 1.0 kb upstream and downstream. *p* values are calculated using Wilcoxon rank-sum tests. **p< 1e-5, ***p< 1e-10.

To understand how RT is epigenetically regulated during differentiation, we compared the four types of RT regions in terms of histone modifications, with focus on H3K4me3 and H3K9me3, together with DNase I hypersensitivity sites (DHSs). H3K4me3 was chosen as a marker of active transcription start sites. H3K9me3 marks Lamin-associated domains (LADs) (Peric-Hupkes et al., 2010), which were highly correlated with late replicating chromatin and the B compartment inHi-C analysis (Rivera-Mulia and Gilbert, 2016). DHSs represent accessible regions, typically regulatory elements on the chromatin. As expected, CE regions were enriched in active mark H3K4me3 (Figure 2B), with strong DHS signals at the TSS (Figure 2D) and were depleted in heterochromatin mark H3K9me3. Meanwhile, the CL regions showed the opposite trend (Figure 2C). These results are consistent with those of previously reported studies (Dileep et al., 2015). The two RT switching categories had similar levels of both types of histone modification before differentiation, in contrast to their opposite RT in hESCs (Figure 2A). In other words, the EtoL and LtoE regions overall were indistinguishable in terms of the three epigenetic features in hESCs. After differentiation, the RT switching was accompanied by concordant changes in histone modifications. Thus, the LtoE regions showed a higher level of H3K4me3, lower H3k9me3, and stronger DHS than the EtoL regions. We observed the same epigenetic features in the four RT domains in the gene body and TSS within 1.0 kb upstream and downstream (Supplementary Figure S4). These results indicated that the changes in status, and not the original status itself, were correlated between RT and histone modifications. The opposite RT switching behavior between EtoL and LtoE cannot be attributed to the histone modifications studied. Thus, we evaluated other aspects of chromatin organization.

### Global 3D chromatin structural features of constitutive and differentiation dynamic replication domains

**FIGURE 3:**
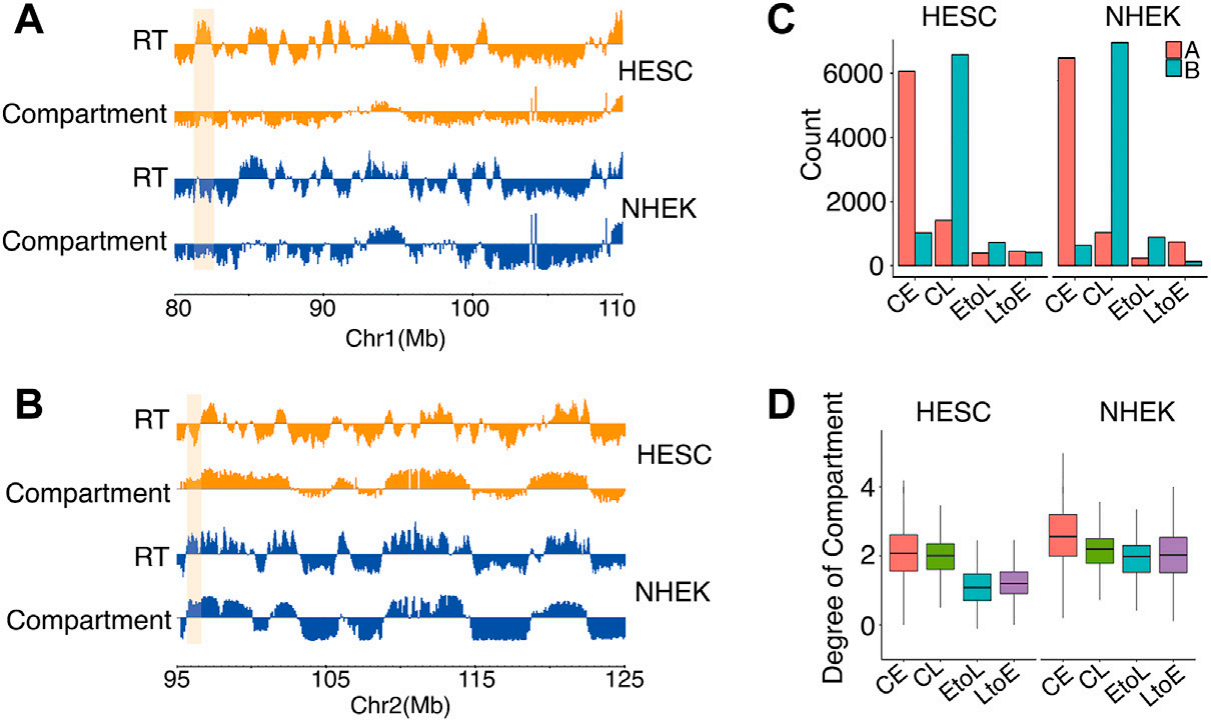
Subnuclear compartment compositions of the four RD categories in hESCs and NHEK cells.**(A,B)** Two examples showing discordance between RT and A/B compartments. The region highlighted in orange shows EtoL in the B compartment in both cell types **(A)**. Region highlighted in orange shows LtoE in the A compartment in both cell types **(B)**.**(C)** Compartment compositions of CE, CL, EtoL, and LtoE in hESC and NHEK cells.**(D)** Degrees of compartmentalization of CE, CL, EtoL,and LtoE in hESC and NHEK cells. *p* values are calculated using the Wilcoxon rank-sum test. ***p < 1e-3.

The bi-partition of the human genome into active and inactive compartments, denoted A and B compartments, respectively, was one of the major findings of the first study applying Hi-C to human cells (Lieberman-Aiden et al., 2009). Replication timing was then reported to be highly correlated with this compartmentation structure (Ryba et al., 2010). Using Hi-C data available for all five cell types, we analyzed the global interaction patterns of the four RT categories and showed the results of hESCs and NHEK cells specifically (Figure 3). As expected, most CE and CL regions were in the A and B compartments, respectively, in hESCs and NHEK cells. This finding indicated the concordant conservation of both RT and structure compartmentalization during cell differentiation. Moreover, the consistency between RT and compartmentalization was higher in differentiated cells than in hESCs. However, the differentiation dynamic RDs showed a more complicated picture. In NHEK cells, both EtoL and LtoE regions have consistent RT and compartment attributes, with good alignment between early RT and A compartment and between late RT and B compartment. However, this is not the case for hESCs, in which the EtoL regions most often occurred in the B compartments, while LtoE regions were more common in A than B compartments. Figures 3A,B presents two examples showing the discordance between RT and compartments in hESC. In both cases, the RT switching is not accompanied by a compartment switch. This discordancy indicates that the dynamic regions already bear structural preferences toward the final RT state rather than the original RT state in hESCs (Figure 3C). As shown in Figure 3D, both dynamic RD categories lie in regions with low degrees of compartmentalization (Materials and Methods), contrary to the increased compartmentalization observed in differentiated cells.

### Local chromatin structural features of constitutive and differentiation dynamic replication domains

**FIGURE 4:**
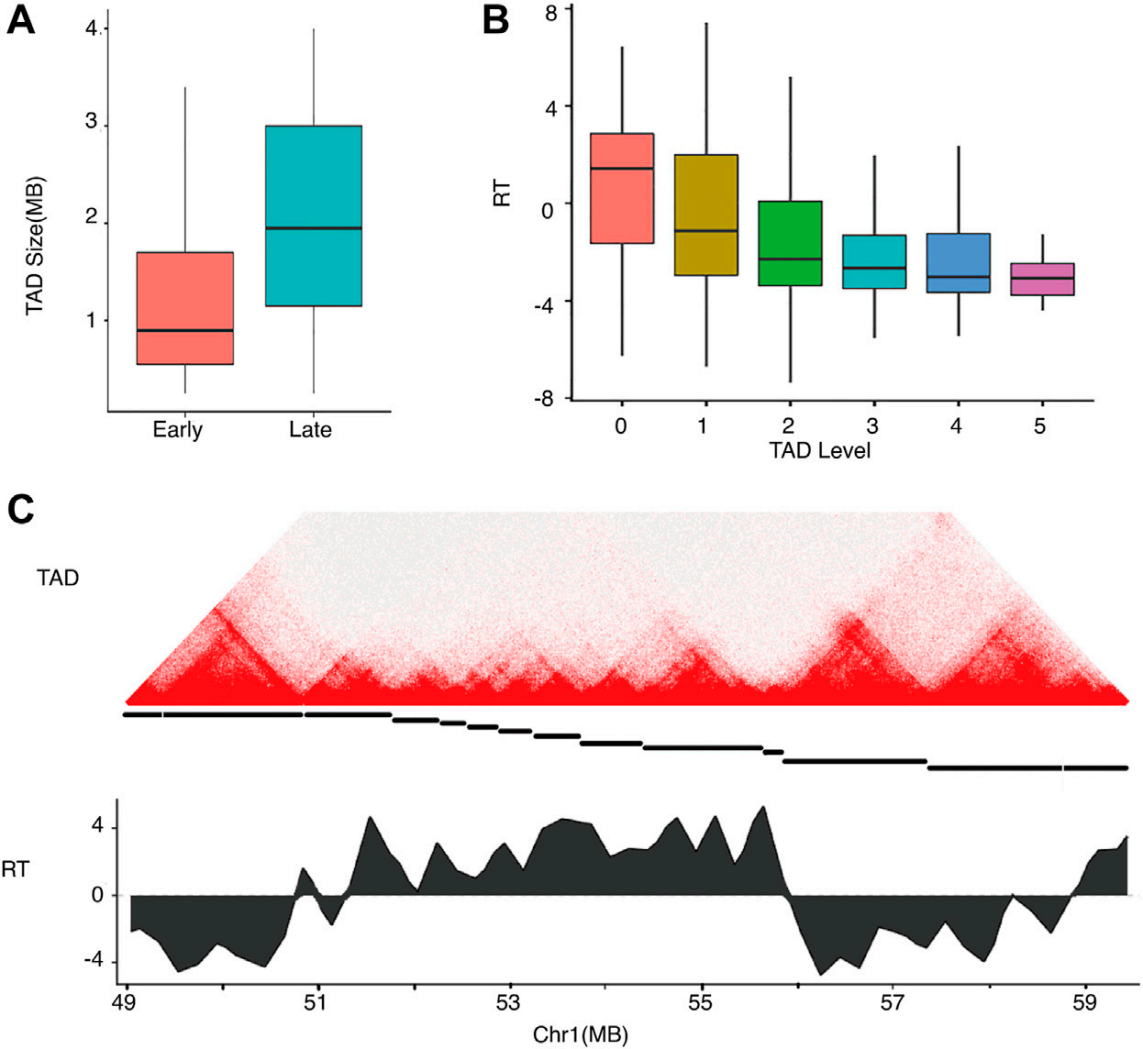
Relationships of replication timing and TAD size and hierarchy in hESC.**(A)** TAD size distributions of early and late RDs.**(B)** RT distributions of TADs in differential levels.**(C)** An example showing a local Hi-C heatmap and RT on chromosome 1. Top: heatmap with colors representing Hi-C contacts. Middle: The TAD structure is denoted by disconnected horizontal lines. Bottom: RT profile for the region showing early and late replication.

Hi-C experiments showed that mammalian genomes are organized into TADs (Dixon et al., 2012) and domains (Rao et al., 2014) at various scales. RDs are highly correlated with TADs (Pope et al., 2014). TADs show hierarchical structures like subTADs (Wijchers et al., 2016) in which a TAD at a lower level may contain multiple subTADs at higher levels. We identified hierarchical TADs using HiTAD (Wang et al., 2017) and compared them to the early and late replicating domains (Figure 4). As shown in Figure 4A, early RDs corresponded to small TADs, while late RDs corresponded to large TADs. These findings are consistent with the observation of smaller early RD sizes than those of late RDs (Rivera-Mulia et al., 2015). The correlation between RT and domain size was expected given the previously observed correlation between chromatin activity and domain size (Hou et al., 2012). Given the identified hierarchical structure, we assigned a level for each domain in HiTAD. TAD was denoted as level 0, subTAD as level 1, and subsequent levels as level 2, 3, etc. As shown in Figure 4B, RT was anti-correlated with the TAD level. DNA in the lowest level of the TAD hierarchy replicates early, while DNA in levels 1 and higher replicated late. A typical early RD corresponded to TADs∼1 mb in size at the lowest level, while a typical late RD corresponded TADs∼2 mb in size at higher levels. Thus, small TADs at a low level of the TAD hierarchy replicate first, followed by larger TADs at higher levels.

**FIGURE 5:**
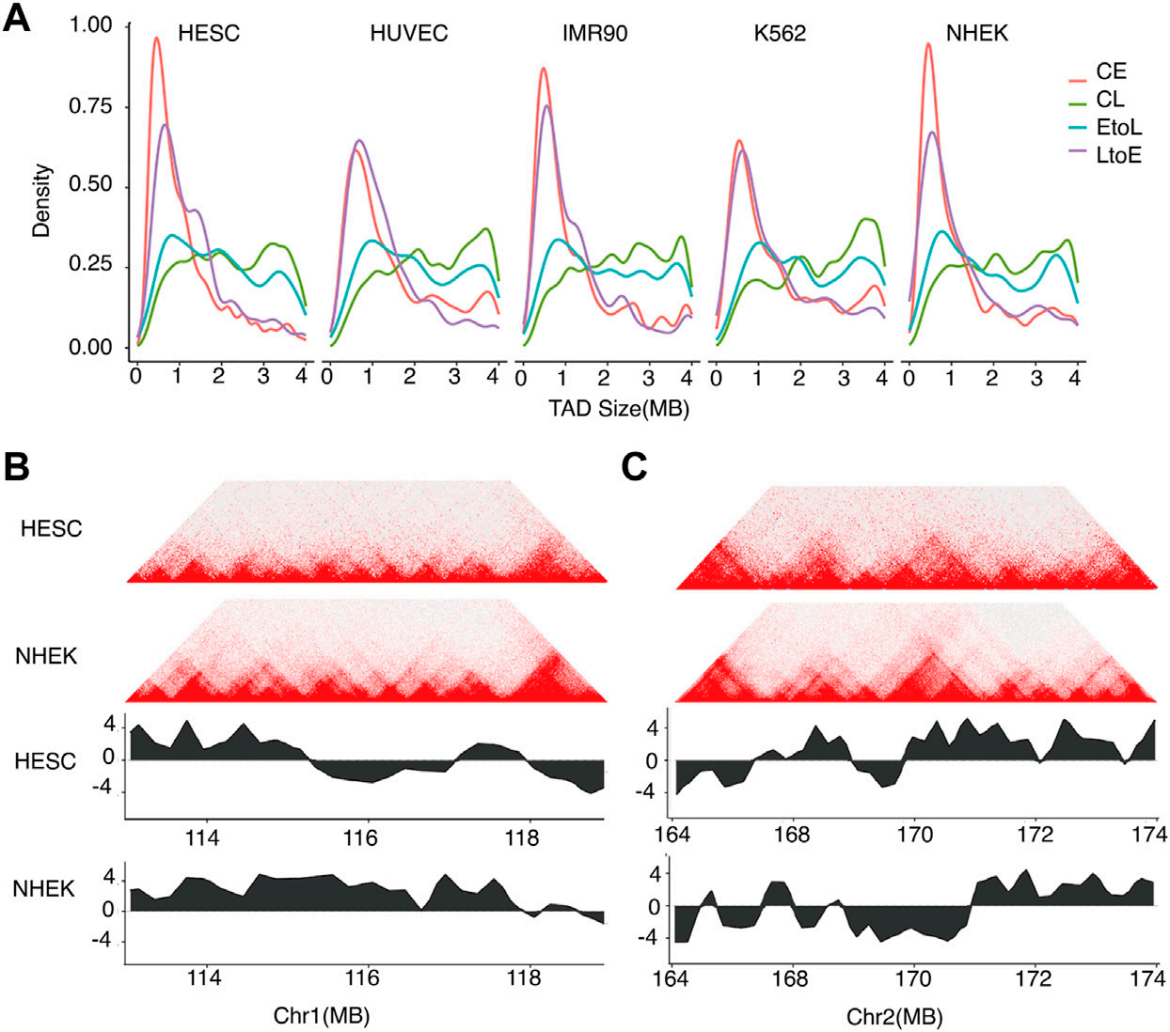
Sizes of TADs corresponding to different types of replication domains.**(A)** TAD size distributions of the four categories of RT regions for the five cell types.**(B)** Typical example of TAD structure of an EtoL region on chromosome 1. Top: Hi-C contact heatmap for hESC and NHEK cells. Bottom: RT profiles for hESC and NHEK cells.**(C)** Typical example of TAD structure of an LtoE region on chromosome 2. Top: Hi-C contact heatmap for hESC and NHEK cells. Bottom: RT profiles for hESC and NHEK cells.

We also compared the TAD size distributions of the four RT categories in each cell type (Figure 5). The overall TAD size distributions of the four RT categories were consistent between different cell types, with CE and CL showing the smallest and largest domain sizes, respectively, and EtoL and LtoE showing intermediate sizes. For the same RT category, the TAD size distributions were quite stable across cell types, implying that TAD size is constant during cell differentiation. More importantly, LtoE regions showed a smaller TAD size distribution than EtoL regions for all cell types assessed in this study. Specifically, LtoE regions mostly occurred in small TADs, while the EtoL regions occurred in large TAD, as shown in the examples in Figures 5B,C. Similar to our findings in compartment analysis, in hESC, the EtoL regions were more similar to CL regions, while the LtoE regions were more similar to CE regions in terms of TAD size.

## Discussion

The replication timing of the human genome is highly dynamic during cell differentiation, with substantial genome turnover between early and late replication. This study aimed to characterize the epigenetic features of dynamic RT regions before and after cell differentiation by integral analysis of histone modifications and chromatin conformation data from multiple cell types. As the 3D genome structure significantly affects the phenotype without altering the DNA sequence, we considered it together with histone modifications as two aspects of epigenetic characteristics. Compared to previous studies on spatiotemporal behavior during the cell cycle or differentiation stages in humans (Boulos et al., 2015; Dileep et al., 2015; Rivera-Mulia et al., 2015; Dileep et al., 2019) and mice (Miura et al., 2019), we focused on the large-scale chromatin structure underlying RT regulation by assessing what structure features facilitated the dedicated and extensive changes of RT program required for differentiation. During differentiation, RT shows diverse and complicated patterns with extensive lineage specificity (Rivera-Mulia et al., 2015). By setting stringent criteria for the consistency of RT behavior between hESCs and the representative cell lines of the three germ layers, we excluded regions with lineage-specific behavior. Thus, we could identify the universal relationship between RT and epigenetic features without confounding due to lineage-dependent differentiation. Comparisons of RT-constitutive and RT-switching regions in hESCs showed that the two RT-switching categories were consistently in intermediate states between constitutive early and constitutive late replication categories. These findings implied that the intermediate, thus flexible and plastic epigenetic environments (Yadav et al., 2018) in terms of histone modifications and degree of compartmentalization, may facilitate RT-switching for proper differentiation.

Intriguingly, our results revealed unexpected epigenetic patterns between early-to-late and late-to-early replication regions. EtoL regions correspond to early replication in hESC; thus, they should show features of active chromatin including in the A compartment and small TAD. In contrast, LtoE regions are late replication in hESC and should show the opposite structural features. However, we observed that in hESC, the EtoL regions appeared more like late-replication regions than LtoE (Figures 3B, 5A). Regarding histone modifications, EtoL and LtoE do not show such opposition and also showed a lack of correlation (Figures 2B,C). Taking together, these findings suggested that the prior acquisition of active chromatin structures in the LtoE region in hESCs indicated a temporal order in which the structural changes preceded the LtoE RT changes. The results from a mouse neural differentiation system (Miura et al., 2019) are consistent with this hypothesis. Combined with the results of previous studies (Jin et al., 2013; Stadhouders et al., 2018) showing pre-induction interactions, the 3D genome architecture tended to be primed to respond to developmental or environmental stimuli.

Given the observed relationship between RT dynamics and chromatin architecture, we speculate a strong connection between large-scale 3D genome structure and the RT program for differentiation. To decipher the causalities, more mechanismic studies are needed. Recently, a CRISPR-based study identified three early replicating control elements (ERCEs) that function cooperatively for the maintenance of pluripotency-associated RD(Sima et al., 2019). This finding implies a scenario in which multiple elements form stable spatial structures to trigger and maintain early replication. Experiments based on the new generation of technologies targeting multiplex interactions (Quinodoz et al., 2018; Zheng et al., 2019) may decipher these mysteries.

While the idea that structure determines function is principally true for proteins, in the context of chromatin in the nucleus of living cells, the relationship between structure and function is more complicated. The introduction of new technologies, especially single-cell sequencing which can resolve challenges with cell heterogeneity, will accelerate our understanding of the interplay between diverse cellular processes and their regulatory mechanisms.

## Data Availability

The original contributions presented in the study are included in the article/Supplementary Material. Further inquiries can be directed to the corresponding author.
