# Supplementary material for: Genome architecture plasticity underlies DNA replication timing dynamics in cell differentiation
Source: Front Genet. 2022 Sep 2;13:961612. doi: 10.3389/fgene.2022.961612 (PMC9478753; doi:10.3389/fgene.2022.961612)
Supplement: Supplementary file 1 [file DataSheet1.docx]

Supplementary Material

Table S1. GEO accession id of the data sets used in this study.

|  | **hESC** | **NHEK** | **IMR90** | **K562** | **HUVEC** |
| --- | --- | --- | --- | --- | --- |
| Repli-seq | GSM923453 | GSM923445 | GSM923447 | GSM923448 | GSM923452 |
| DNase-seq | GSM736582 | GSM816635 | GSM100858 | GSM736629 | GSM816646 |
| Hi-C | GSM230902 | GSE63525 | GSE63525 | GSE63525 | GSE63525 |
| H3K4me3 | GSM733657 | GSM733720 | GSM469970 | GSM733680 | GSE96250 |
| H3K9me3 | GSM605328 | GSM100352 | GSM521913 | GSM733776 | GSM100351 |
| ChIP Control | GSM733770 | GSM733740 | GSE16256 | GSM733780 | GSM733715 |

Table S2 Basic information of Repli-seq data

| **Cell** | **Different Stages** | **Total Reads** | **Uniquely Mapped** | **Mapping ratio** |
| --- | --- | --- | --- | --- |
| HECS | S1+S2 | 9,665,092 | 4,789,854 | 0.4956 |
|  | S3+S4 | 9,412,489 | 4,005,269 | 0.4255 |
| NHEK | S1+S2 | 31,734,237 | 14,977,234 | 0.4720 |
|  | S3+S4 | 30,483,311 | 14,049,958 | 0.4609 |
| IMR90 | S1+S2 | 38,122,024 | 22,078,645 | 0.5792 |
|  | S3+S4 | 39,436,676 | 21,185,433 | 0.5372 |
| K562 | S1+S2 | 6,548,671 | 2,690,326 | 0.4108 |
|  | S3+S4 | 7,417,488 | 3,892,517 | 0.5248 |
| HUVEC | S1+S2 | 15,754,258 | 10,973,295 | 0.6965 |
|  | S3+S4 | 24,439,349 | 16,891,474 | 0.6912 |

Table S3 Basic information of ChIP-seq and DNase-seq data

|  | **Cell** | **Total Reads** | **Uniquely Mapped** | **Mapping Ratio** |
| --- | --- | --- | --- | --- |
| H3K4me3 | HESC | 16,475,284 | 11,704,027 | 0.7104 |
|  | NHEK | 20,855,214 | 11,029,204 | 0.5288 |
|  | IMR90 | 40,385,339 | 28,197,920 | 0.6982 |
|  | K562 | 25,272,278 | 14,479,778 | 0.5730 |
|  | HUVEC | 61,420,537 | 45,405,130 | 0.7392 |
| H3K9me3 | HESC | 20,571,273 | 14,869,906 | 0.7228 |
|  | NHEK | 40,023,596 | 23,951,146 | 0.5984 |
|  | IMR90 | 19,579,676 | 16,144,897 | 0.8246 |
|  | K562 | 46,226,003 | 30,987,461 | 0.6703 |
|  | HUVEC | 50,404,820 | 14,308,262 | 0.2839 |
| control | HESC | 19,775,459 | 10,130,860 | 0.5123 |
|  | NHEK | 21,067,457 | 10,134,493 | 0.4810 |
|  | IMR90 | 15,316,473 | 11,029,391 | 0.7201 |
|  | K562 | 27,579,809 | 18,093,078 | 0.6560 |
|  | HUVEC | 19,523,271 | 12,021,125 | 0.6157 |
| DNase-seq | HESC | 33,448,473 | 24,431,579 | 0.7304 |
|  | NHEK | 53,555,638 | 38,238,113 | 0.7140 |
|  | IMR90 | 277,303,732 | 177,303,932 | 0.6394 |
|  | K562 | 58,028,122 | 35,811,563 | 0.6171 |
|  | HUVEC | 40,580,317 | 28,603,106 | 0.7049 |

Table S4 Basic information of Hi-C data

| **cell** | **Total Reads** | **Total Contacts** |
| --- | --- | --- |
| HESC | 312,782,126 | 186,856,095 |
| NHEK | 1,073,207,572 | 664,899,299 |
| IMR90 | 1,848,905,980 | 1,136,673,290 |
| K562 | 3,610,807,849 | 932,208,867 |
| HUVEC | 6,532,921,401 | 460,393,495 |

**
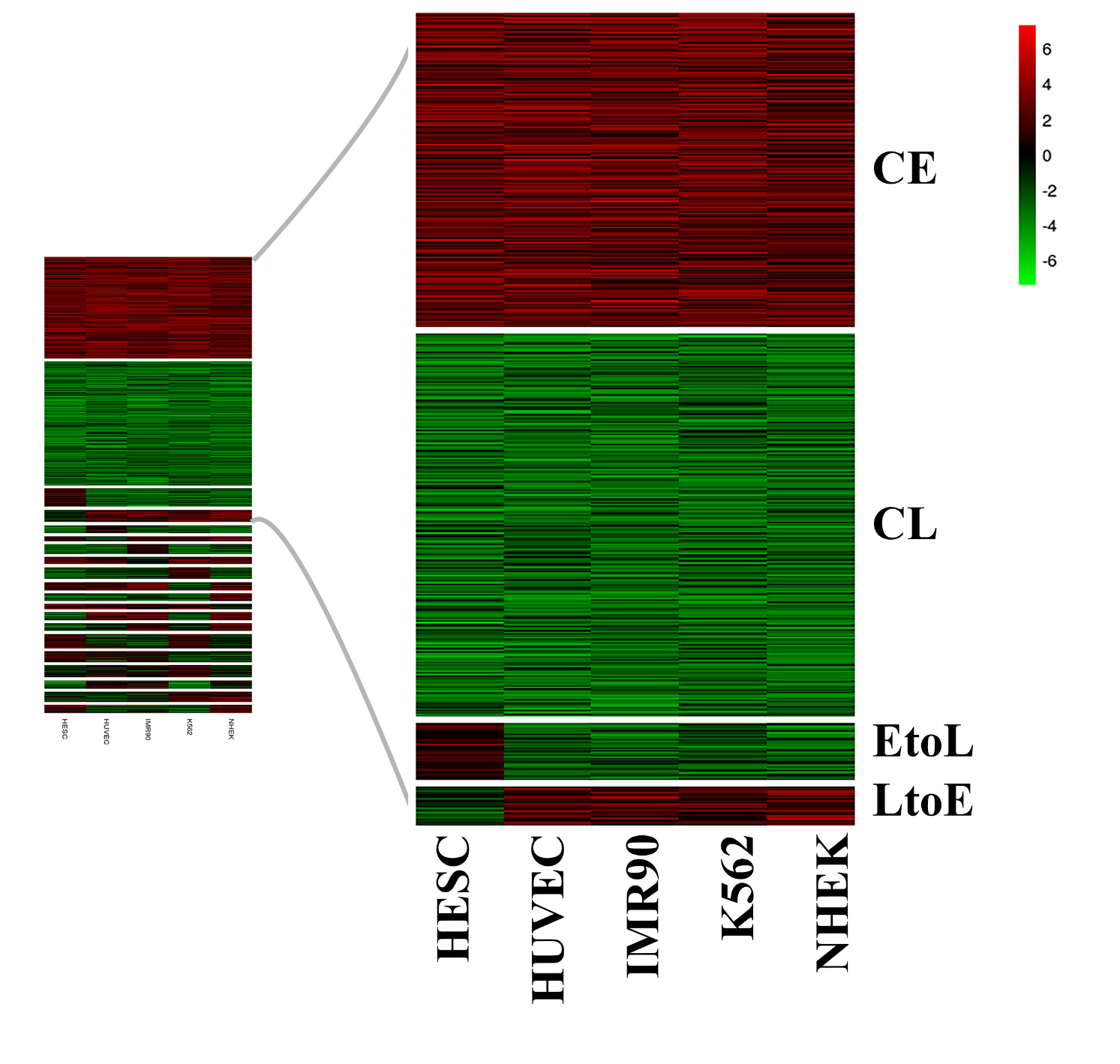
**

Figure S1. Heatmap showing the replication timing profiles of the four categories of genomic bins for the five cell types. Number of bins are 7,142 for CE, 8,700 for CL, 1,299 for EtoL and 881 for LtoE. 28479


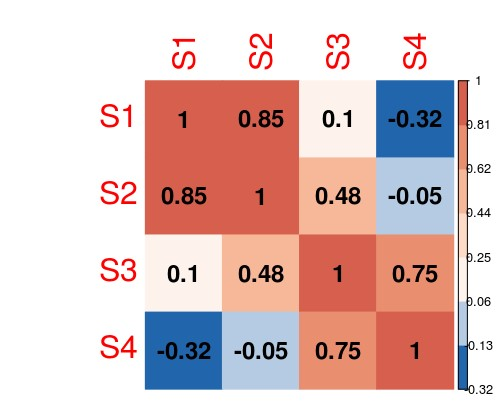


Figure S2. Pearson correlation of repli-seq data of phase S1-S4.

**
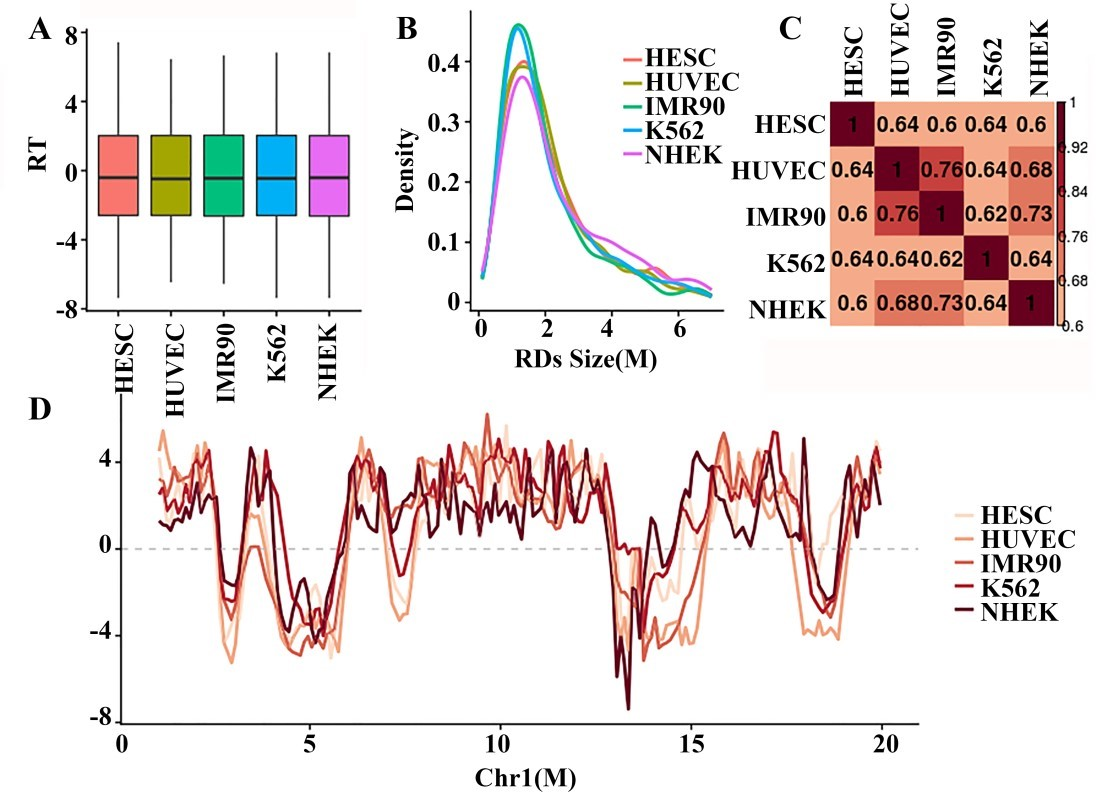
**

Figure S3. Overview of replicating timing data in the five cell types. A. Distribution of RT. B. Distribution of replication domain size. C. Spearman correlation coefficients of the five cell types. D. Example of a typical region on chromosome 1 showing replicating timing profiles in the five cell types.


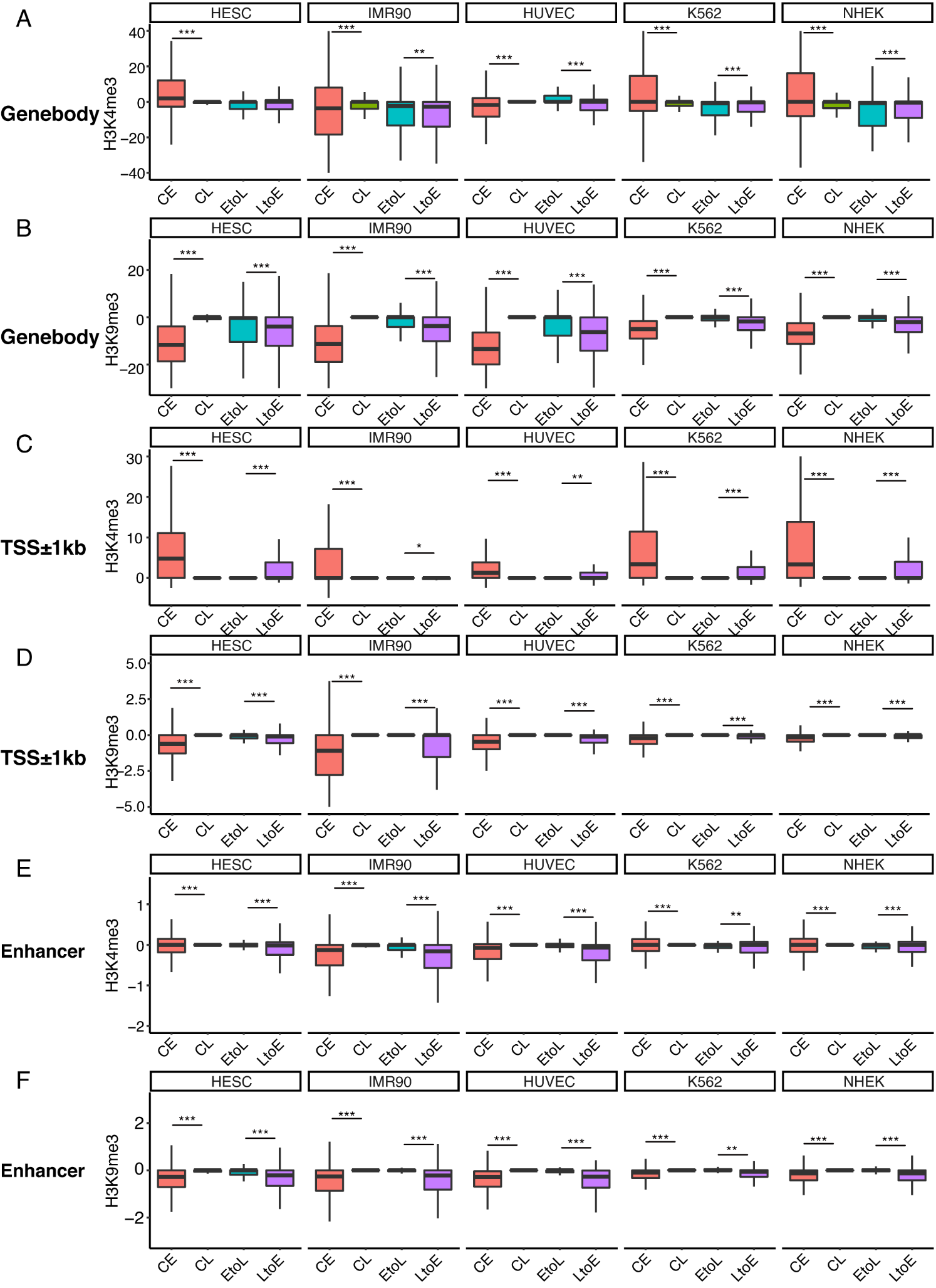


Figure S4. Histone modification of the four RT classes in genebody, TSS±1kb and enhancer region. (A) H3K4me3 signal distribution of the four replication domains in genebody: constitutive early (CE), constitutive late (CL), early-to-late (EtoL), late-to-early (EtoL). CE and CL regions are significantly different in all cell types. EtoL and LtoE regions show significantly different signals in differentiated cells. (B) H3K9me3 signal distribution in genebody. CE and CL regions are significantly different in all cell types studied. EtoL and LtoE regions show significantly different signals in differentiated cells. (C) H3K4me3 signal distribution of the four replication domains in TSS±1kb region: constitutive early (CE), constitutive late (CL), early-to-late (EtoL), late-to-early (EtoL). CE and CL regions are significantly different in all cell types. EtoL and LtoE regions show significantly different signals in differentiated cells. (D) H3K9me3 signal distribution in TSS±1kb region. CE and CL regions are significantly different in all cell types studied. EtoL and LtoE regions show significantly different signals in differentiated cells. (E) H3K4me3 signal distribution of the four replication domains in enhancer: constitutive early (CE), constitutive late (CL), early-to-late (EtoL), late-to-early (EtoL). CE and CL regions are significantly different in all cell types. EtoL and LtoE regions show significantly different signals in differentiated cells. (F) H3K9me3 signal distribution in the enhancer. CE and CL regions are significantly different in all cell types studied. EtoL and LtoE regions show significantly different signals in differentiated cells. P values are calculated using Wilcoxon rank sum test. * p < 5e-2, ** p < 1e-2, *** p < 1e-3.
